# FISH improves risk stratification in acute leukemia by identifying *KMT2A* abnormal copy number and rearrangements

**DOI:** 10.1038/s41598-022-13545-y

**Published:** 2022-06-10

**Authors:** Qinlu Li, Shugang Xing, Heng Zhang, Xia Mao, Min Xiao, Ying Wang

**Affiliations:** Department of Hematology, Tongji Hospital, Tongji Medical College, Huazhong University of Science and Technology, Wuhan, 430030 China

**Keywords:** Genetics, Biomarkers, Molecular medicine, Oncology, Risk factors

## Abstract

Most cases of acute leukemia (AL) with *KMT2A* rearrangement (*KMT2A*-r) have a dismal prognosis. Detection of this aberration in Chinese adult patients relies on reverse transcription polymerase chain reaction (RT-PCR) and chromosome banding analysis (CBA). The fluorescence in situ hybridization (FISH) probe for *KMT2A* detects *KMT2A*-r and copy number variation (CNV) but is not routinely used as a detection technique. This study investigated the potential value of FISH in the treatment of AL by performing FISH along with CBA and RT-PCR in 269 de novo cases of AL. The three detection techniques were compared in identification of *KMT2A*-r, and the applicability of FISH for detecting *KMT2A* CNV was evaluated. Twenty-three samples were identified as positive for *KMT2A*-r (20 using FISH, 15 using RT-PCR, 16 using CBA, and eight according to all three). FISH also identified 17 *KMT2A* CNV, 15 with gains and two with deletions. Ten patients with acute myeloid leukemia (AML) harboring *KMT2A* CNV had a complex karyotype, a negative prognostic factor in AML. Adding FISH of *KMT2A* to routine detection leads to more accurate detection of *KMT2A*-r and improved identification of *KMT2A* CNV, which would benefit patients by improving the risk stratification in AL.

## Introduction

Translocations involving the lysine (K)-specific methyltransferase 2A gene (*KMT2A*), previously known as mixed lineage leukemia (*MLL*), are frequently associated with acute lymphoid leukemia (ALL), acute myeloid leukemia (AML), and mixed-phenotype acute leukemia (MPAL)^1^. According to the World Health Organization (WHO) 2016 classification, leukemia with *KMT2A* rearrangements (*KMT2A*-r) is an independent subtype that often leads to a dismal prognosis, except for the intermediate prognostic factor t(9;11) involving *KMT2A-MLLT3*^2^. *KMT2A*-chimeric genes that remain stable during clonal evolution of tumors can be used for molecular monitoring of minimal residual disease, which is a reliable predictor of clinical outcome^3^. Accordingly, timely and accurate identification of *KMT2A* aberrations is of great clinical significance. To date, a total of 135 types of *KMT2A*-r have been identified, of which 94 translocation partner genes have been characterized at the molecular level^4^. In addition to the wide range of partner genes, the location of the *KMT2A* breakpoint varies within different chimeric genes^5^. Therefore, confirmation of chromosomal rearrangements in *KMT2A*-associated acute leukemia (AL) remains a difficult diagnostic task owing to the high heterogeneity of the rearrangements.

Currently, chromosomal banding analysis (CBA) and commercial kits in multiplex reverse transcription polymerase chain reaction (RT-PCR) are routinely used in China as the main methods of detecting *KMT2A*-r. However, both methods have inherent limitations. CBA may miss *KMT2A*-r, possibly owing to the low mitotic index of leukemic cells and the presence of complex or cryptic chromosomal abnormalities^6^. Meanwhile, multiplex RT-PCR kits can detect only a limited number of the most frequent *KMT2A*-r^7^. A commercially available dual-color fluorescence in situ hybridization (FISH) break apart probe is only used as an auxiliary method to detect *KMT2A*-r when there is a discrepancy in CBA and RT-PCR results; there is a lack of comprehensive understanding of *KMT2A*-r heterogeneity and widespread underestimation of the value of the FISH test.

Although uncommon, amplification of the 11q23 region is recognized as a recurrent event in both AML and myelodysplastic syndrome (MDS) and is associated with older age, a complex karyotype (CK), and a very poor prognosis^8–10^. In addition to detecting *KMT2A*-r, the *KMT2A* probe can detect the copy number variation (CNV) of *KMT2A*. However, we are unaware of any studies that aimed to elucidate the value of the FISH technique in the identification of both rearrangement and numerical abnormality of *KMT2A*. In this study, FISH analysis of *KMT2A* was performed in each newly diagnosed adult patient with AL at a single institution. The study analyzed the distribution of *KMT2A*-r in Chinese adults with AL using a combination of different testing methods, including RT-PCR, CBA, and FISH, and it evaluated the clinical significance of including the *KMT2A* probe in *KMT2A* CNV detection methods in routine diagnostic workups.

## Materials and methods

### Patients

A total of 269 patients with de novo AL from Tongji Hospital at Tongji Medical College of Huazhong University of Science and Technology (Wuhan, China) between January 2019 and November 2020 were selected for the study. All cases were categorized according to the 2016 WHO classification^2^. Bone marrow (BM) or peripheral blood (PB) samples were collected from the patients at diagnosis, and their medical records were retrospectively reviewed.

### Conventional cytogenetics analysis

CBA of BM or PB cells was performed using short-term (24 h) and unstimulated cultures, according to standard G-banding procedures. The G-banded metaphase chromosomes were obtained by treatment of the cells with trypsin, and they were then stained with Leishman’s stain. At least 20 metaphase cells were analyzed in detail for each sample. Karyotypes were interpreted according to the 2016 International System for Human Cytogenetic Nomenclature^11^.

### FISH analysis

FISH analysis for *KMT2A*-r was performed using fixed cells and the *LSI KMT2A* dual-color, break apart rearrangement probe (BAP; Abbott Laboratories, Abbott Park, IL, USA) according to the manufacturer’s instructions. A minimum of 200 nuclei or metaphase chromosomes per sample were evaluated for *KMT2A*-r. The probe produced two fusion signals of orange and green when hybridized to normal nuclei; green (5′-*KMT2A*), orange (3′-*KMT2A*), and orange/green fusion signals were detected in nuclei containing typical *KMT2A*-r. Cases with either a single copy or ≥ 3 *KMT2A* copies were considered as numerically abnormal *KMT2A*.

### RT-PCR

The RNA samples were prepared from mononuclear cells using a RNeasy Mini Kit (Qiagen, Redwood City, CA, USA) according to standard protocols. Next, RT-PCR was conducted for the 43 fusion genes (FGs) using a Leukemia-Related Fusion Gene Detection Kit (Yuanqi Bio-Pharmaceutical Co., Ltd., Shanghai, China) according to the manufacturer’s instructions. The screening panel tested for partial tandem duplications (PTD) specific for *KMT2A* (*KMT2A*-PTD) and 10 relatively common types of FGs involving *KMT2A* (*KMT2A*-FG): *KMT2A*-*MLLT10*, *KMT2A*-*MLLT6*, *KMT2A*-*EPS15*, *KMT2A*-*MLLT11*, *KMT2A*-*AFF1*, *KMT2A*-*MLLT4*, *KMT2A*-*MLLT3*, *KMT2A*-*SEPT6*, *KMT2A*-*ELL*, and *KMT2A*-*MLLT1*.

### Flow cytometric immunophenotyping

After RT-PCR, PB or BM blast cells were analyzed using flow cytometry. A panel of monoclonal antibodies against CD45, CD34, CD38, CD33, CD56, CD3, CD2, CD5, CD7, CD10, CD8, CD19, CD20, CD138, CD24, CD22, CD28, kappa, lambda, TdT, HLA-DR, and CD79a was used as previously described to determine the immunophenotypes of the leukemia cells^12^.

### Statistical analysis

IBM SPSS Statistics software (version 16.0; SPSS Inc., Chicago, IL, USA) was used to perform all statistical analyses. Comparisons between groups were performed using χ2 tests. The statistical significance was set at *P* < 0.05.

### Compliance with ethical standards

Appropriate informed consent was obtained from all donors prior to specimen collection in accordance with the Declaration of Helsinki and the research protocol approved by the ethics committees of Tongji Hospital.

## Results

### Patient characteristics

In total, 269 newly diagnosed adult AL patients were evaluated. The median patient age was 45 years (range 18–86) and the male: female ratio was 1.56 (164:105). Of the 269 patients, 185 had AML, 74 had ALL, and 10 had MPAL. A total of 23 cases exhibited *KMT2A-*r based on a combination of cytogenetic and molecular techniques, including FISH, CBA, and specific RT-PCR methods. Of the 23 *KMT2A*-r cases, samples from eight patients were positive according to all three tests. Meanwhile, 17 cases were identified as having *KMT2A* CNV. The clinicopathological features of the 269 AL patients are listed in Table 1.

**Table 1:** The clinical and pathological features of the 269 AL patients.

Variable	Number of cases	Percentage
**Age**		
18–40	107	39.8
41–60	95	35.3
≥ 60	67	24.9
**Sex**		
Male	164	61.0
Female	105	39.0
**Diagnosis**		
AML	185	68.8
ALL	74	27.5
MPAL	10	3.7
**Cytogenetic abnormality**		
KMT2A rearrangement	23	8.6
KMT2A copy number variation	17	6.3
Complex karyotype	37	13.8

### FISH analysis

**Figure 1:**
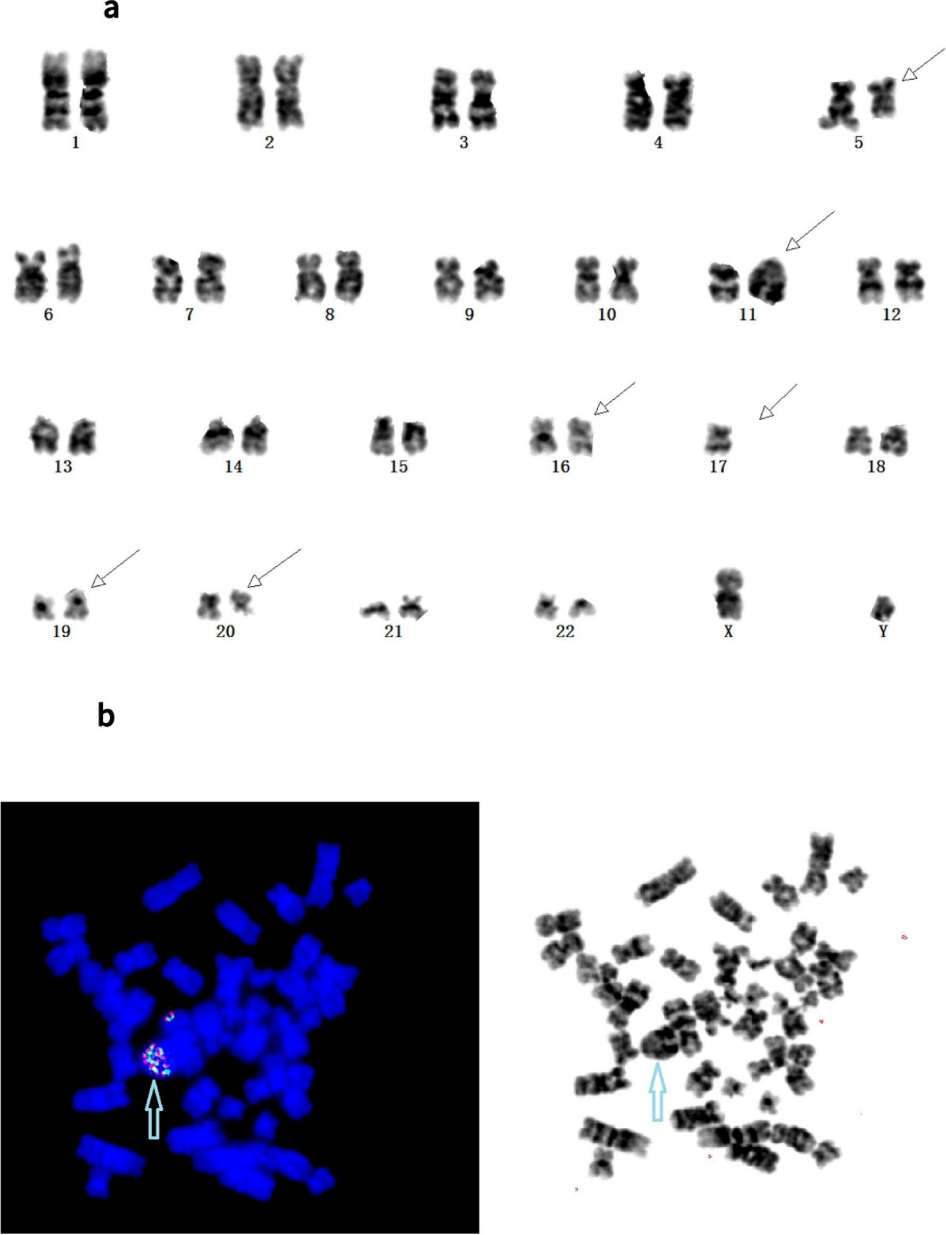
(**a**) Chromosome banding analysis of patient 25 showing a complex karyotype: 45,XY,del(5)(q13q32),del(11)qdp(11)(q21q23)r(11)(p15;q25),der(16;17)(p10;q10),add(19)(q13),del(20)(q11). (**b**) Fluorescence in situ hybridization (FISH) analysis of Patient 25 using the *KMT2A* probe revealed multiple *KMT2A* fusion signals present on derivative chromosome 11 (blue arrow).

FISH analysis revealed that 37 of the 269 AL patients (14.9%) exhibited aberrant *KMT2A* signals, including 20 cases with a *KMT2A*-r signal (14 with AML, 5 with ALL, and 1 with MPAL). The samples with a *KMT2A*-r signal included 17 cases of typical positive signals and three cases of atypical abnormal signals (Patient 20: one fusion and one green signal, Patient 3: two fusion signals with one exhibiting a diminished red signal, and Patient 2: two fusion signals with one extra green signal). The clinical and laboratory characteristics of these patients are listed in Table 2. The remaining 17 cases had *KMT2A* CNV, including multiple copy numbers of *KMT2A* in 15 patients and a single copy in the other two patients. As shown in Fig. 1, the multiple copy numbers of *KMT2A* varied, with three copies in each of seven cases (Patients 31, 32, 33, 34, 35, 36, and 40), four copies in four cases (Patients 28, 29, 30, and 39), five copies in two cases (Patients 26 and 27), and more than five copies in two cases (Patients 24 and 25). The clinical and laboratory characteristics of these patients are listed in Table 3.

**Table 2:** Clinical, cytogenetic and molecular results of 23 patients with *KMT2A* rearrangements.

Patient number	Sex	Age	Diagnosis	Karyotype	FISH signal	Fusion transcript
1	M	19	AML	49,XY, + 19, + 21, + add(21)(p11) [10]	1F1R1G	N
2	F	22	AML	46,XX [20]	2F1G	KMT2A-MLLT4
3	F	25	AML	46,XX [25]	2F(1F with diminished red signal)	KMT2A-MLLT4
4	M	30	AML	46,XY [20]	N	KMT2A-SEPT6
5	F	72	AML	46,XX [20]	N	KMT2A-PTD
6	M	41	AML	51,XY, + 3, + 6, + 8, + 18, + 19[7] /46,XY [13]	N	KMT2A-MLLT10
7	M	52	AML	failure	1F1R1G	KMT2A-MLLT1
8	M	46	AML	47,XY, + 3,t(10;11)(p12;q22) [8]/46,XY [2]	1F1R1G	N
9	F	52	AML	46,XX,t(2;11)(p21;q23) [15]	1F1R1G	N
10	F	62	AML	48,XX, + 8, + 11,t(5;11)(q31;q23) [10]	2F1R1G	N
11	M	21	AML	46,XY,t(11;19)(q23;p13.1) [9]/46,XY [11]	1F1R1G	N
12	F	62	AML	50,XX, + 8, + add(9)(p24),t(11;17)(q23;q25), + 13, + 21 [20]	1F1R1G	N
13	M	32	AML	46,XY,t(11;17)(q23;q21) [5]/46,XY [8]	1F1R1G	N
14	M	18	ALL	46,XY,t(4;11)(q21;q23) [9]/46,XY [1]	1F1R1G	N
4	F	50	ALL	46,XX,t(11;19)(q23;p13.3) [10]	1F1R1G	KMT2A-MLLT1
16	M	49	ALL	47,XY, + X,t(11;19)(q23;p13.3) [5]/46,XY [5]	1F1R1G	KMT2A-MLLT1
17	M	49	AML	46,XY,t(6;11)(q27;q23) [8]/46,XY [2]	1F1R1G	KMT2A-MLLT4
18	F	22	AML	46,XX,t(9;11)(p21;q23) [5]/47,idem, + 8 [5]	1F1R1G	KMT2A-MLLT3
19	F	26	AML	46,XX,t(11;19)(q23;p13.1) [8]	1F1R1G	KMT2A-ELL
20	M	28	AML	46,XY,t(11;19)(q23;p13.1) [6]/46,XY [5]	1F1G	KMT2A-ELL
21	M	18	MPAL	46,XY,t(9;11)(p21;q23) [5]/46,XY [5]	1F1R1G	KMT2A-MLLT3
22	F	54	ALL	47,XX, + X,t(1;11)(p32;q23) [10]	1F1R1G	KMT2A-EPS15
23	M	31	ALL	47,XY,add(1)(p36),t(4;11)(q21;q23), + 8 [10]	1F1R1G	KMT2A-AFF1

*F* female, *M* male, *ALL* acute lymphoid leukemia, *AML* acute myeloid leukemia, *MPAL* mixed-phenotype acute leukemia, *N* negative.

**Table 3:** Clinical, cytogenetic and molecular results of 17 patients with *KMT2A* copy number variation.

Patient number	Sex	Age	Diagnosis	FISH	Gene	Karyotype
24	F	56	AML	> 5F	N	46,XX,add(1)(p36),del(3)(p11),add(5)(p15),add(5)(q23),r(11)(p15q25), del(13)(q21),add(14)(q23),del(15)(q22) [9]/46,XX [9]
25	M	79	AML	> 5F	N	45,XY,del(5)(q13q32),der(11)qdp(11)(q21q23)r(11)(p15;q25), der(16;17)(p10;q10),add(19)(q13),del(20)(q11) [10]
26	M	61	AML	5F	N	41–44,XY,del(2)(p13),-4,del(5)(q13), -7,add(9)(q34),?add(19)(q13),del(20)(q11),-22, + mar[cp7]/46,XY [2]
27	F	72	AML	5F	N	failure
28	F	28	AML	4F	N	45,XX,del(5)(q11q21),del(6)(q11),-7,add(8)(q24),add(12)(p11) [14]/46,XX [1]
29	M	73	AML	4F	N	47,XY, + mar [2]
30	F	60	AML	4F	N	60,XX,-X,-3,-4,del(5)(q21),del(5)(q21),-6,-7,-9, + 11, -12,-14,-17,-21[cp13]
31	M	65	AML	3F	N	47,XY, + 11,i(17)(q10),?del(16)(q11) [10]
32	M	27	AML	3F	N	53,XY, + 4,del(6)(q22), + 9, + 11, + 13, + 19, + 21, + 21 [3]/46,XY [4]
33	M	30	AML	3F	N	49–51,XY, + del(3)(p11), + 4, + del(6)(q23), + del(11)(q24), + 14,i(17)(q10)[cp10]
34	F	66	AML	3F	N	44–45,XX,del(3)(p21),del(5)(q21),?-7, t(12;14)(p10;q10),add(10)(q25)[cp6]
35	M	63	AML	3F	N	47,XY, + 11 [8]/46,XY [2]
36	F	75	AML	3F	N	47,XX, + 11 [10]
37	M	38	AML	1F	N	46,XY,-2,add(11)(q23), + mar [10]
38	F	76	AML	1F	N	46,XX,add(11)(q23) [5]/46,idem,del(5)(q22q34) [5]
39	M	18	ALL	4F	N	56–59,X, + X,-Y, + 1, + 5, + 6, + 7, + 8, + 8, + 10, + 11, + 11, + 14, + 19, + 21, + 21[cp10]
40	F	30	ALL	3F	N	47,XX,add(9)(q34), + 11 [2]/46,XX [11]

*F* female, *M* male, *ALL* acute lymphoid leukemia, *AML* acute myeloid leukemia, *N* negative, *F* fusion.

### Conventional cytogenetic results

Successful CBA was obtained in 255 patients. A total of 120 (44.6%) patients demonstrated an abnormal karyotype, including 16 (6.3%) cases with *KMT2A*-r (10 AML, 5 ALL, and 1 MPAL). Of the 185 AML cases, 28 (15.1%) showed CK with at least three chromosomal abnormalities, including 10 that had *KMT2A* CNV identified via FISH analysis. The incidence rate of CK in the group with *KMT2A* CNV was significantly higher than that in the group with a normal *KMT2A* copy number (10/15 vs. 18/170, respectively, *P* < 0.01; Table 4). Six patients of CK with *KMT2A* CNV had either del (5q) or other structural aberrations involving 5q. Among the 74 ALL patients, CK was detected in nine cases without numerical abnormality of KMT2A.

**Table 4:** The results of KMT2A copy number and complex karyotype in AML.

	CK	Non-CK	Total	p
KMT2A CNV	10 (66.7%)	5 (33.3%)	15	< 0.01
KMT2A CNN	18 (10.6%)	152 (89.4%)	170	

*KMT2A CNV* KMT2A copy number variation, *KMT2A CNN* normal KMT2A copy number, *CK* complex karyotype.

### Metaphase FISH after karyotype

**Figure 2:**
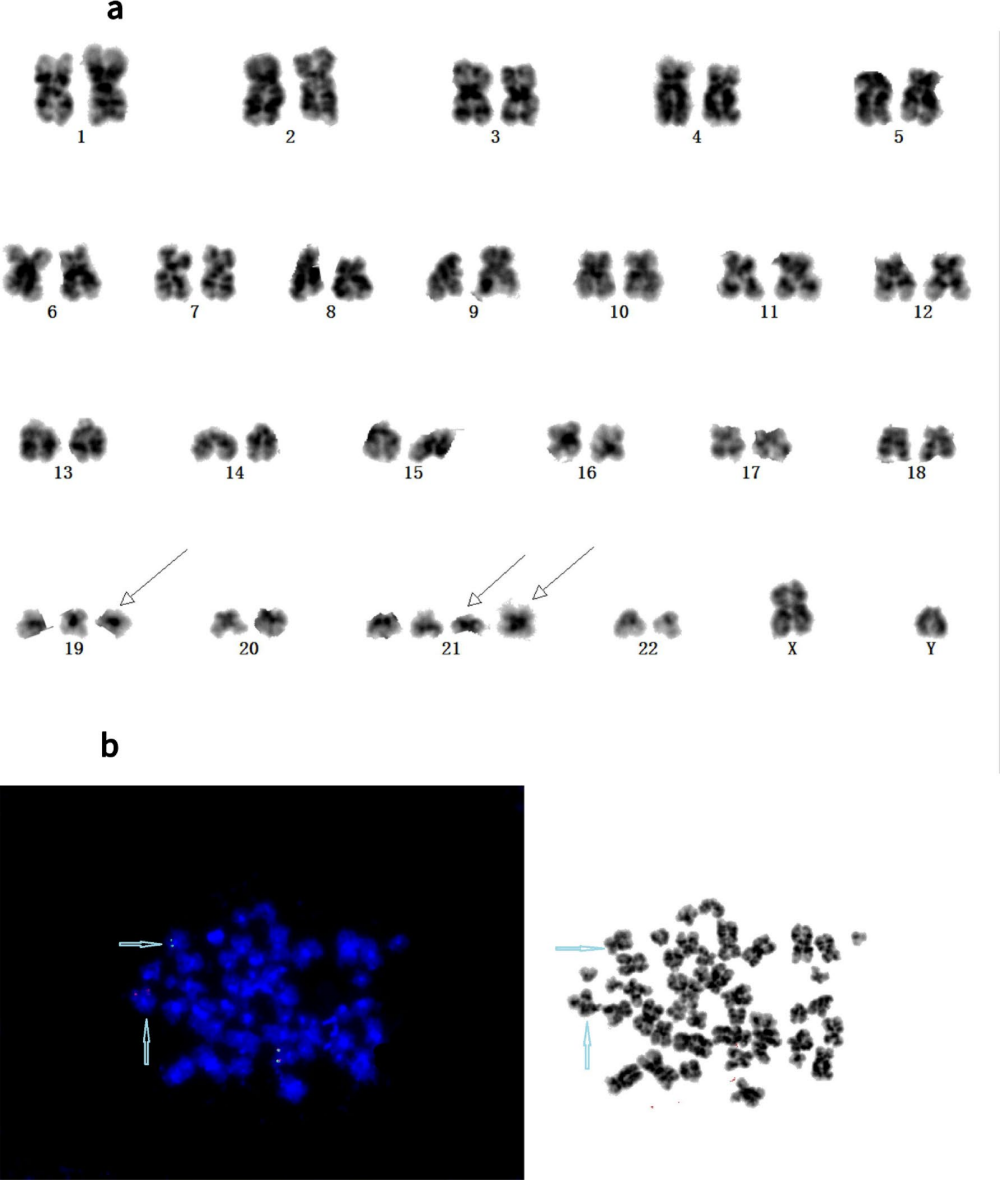
(**a**) Karyotype of Patient 1 showing 49,XY, + 19, + 21, + add(21)(p10). (**b**) Metaphase fluorescence in situ hybridization (FISH) analysis of patient 1 using the *KMT2A* probe, with chromosome 11 presenting a normal *KMT2A* gene signal (fusion-yellow FISH signal), a translocation presenting splatted signals, a green *KMT2A* signal in the short arm of a derivative chromosome 10, and an orange signal indicating the long arm of derivative chromosome 11 (blue rows).

**Figure 3:**
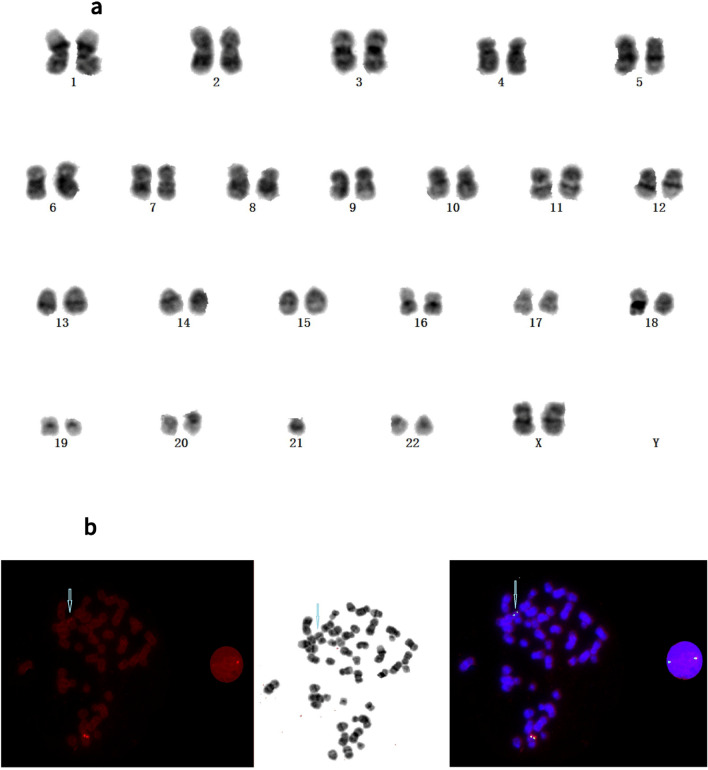
(**a**) Karyotype of Patient 3 showing 46,XX. (**b**) Fluorescence in situ hybridization (FISH) analysis of a metaphase cell of Patient 3 using the *KMT2A* probe. Two intact *KMT2A* copies were revealed, and one of the two *KMT2A* fusion signals (3′-*KMT2A* orange signal) was significantly diminished (blue arrow).

Repeated G-banding karyotype analysis (with the assistance of the metaphase FISH of *KMT2A* analysis) confirmed three additional cases harboring atypical *KMT2A*-r (Patients 1, 2, and 3). Initially, Patient 1 showed no structural or numeric abnormalities involving chromosomes 10 or 11 and typical rearrangement signals of one fusion, one green, and one orange signal in the nuclei. However, metaphase FISH analysis using the *KMT2A* BAP showed a 5′-green signal on the short arm of chromosome 10, whereas the 3′-orange signal of the *KMT2A* probe on the long arm of chromosome 11 was retained. Therefore, submicroscopic insertion of the 5′ region of *KMT2A* into 10p had occurred (Fig. 2). The RT-PCR failed to detect the *KMT2A-MLLT10* fusion transcript in leukemic cells, most likely because of inappropriate primers being used. Meanwhile, Patient 2 had a normal karyotype but showed the atypical result of an extra 5′-*KMT2A* green signal in interphase FISH analysis using *KMT2A* BAP. FISH analysis following metaphase revealed that the extra 5′-*KMT2A* green signal was located in the long arm of chromosome 6 and was due to the partial duplication and insertion of *KMT2A* into *MLLT4,* resulting in the *KMT2A*-*MLLT4* FG-positive finding. Karyotype analysis of Patient 3 showed an initial normal karyotype. However, *KMT2A* BAP FISH analysis revealed two intact *KMT2A* FISH signals, with one of the two *KMT2A* fusion signals demonstrating a significantly diminished 3′-*KMT2A* orange signal in nuclei during metaphase (Fig. 3), suggesting an atypical *KMT2A*-r.

### RT-PCR results

Among the 269 patients, there were 15 cases that were multiplex RT-PCR–positive for FGs produced by *KMT2A*-r, including one each of *KMT2A*-*AFF1*, *KMT2A*-*EPS15*, *KMT2A*-*SEPT6*, *KMT2A*-*PTD*, and *KMT2A*-*MLLT10*; two each of *KMT2A*-*MLLT3* and *KMT2A*-*ELL*; and three each of *KMT2A*-*MLLT1* and *KMT2A*-*MLLT4*. Of the 15 positive cases, *KMT2A* FISH and karyotype analyses failed to reveal any aberration involving *KMT2A*-r in Patients 4, 5, and 6, who were positive for *KMT2A*-*SEPT6*, *KMT2A*-*PTD*, and *KMT2A*-*MLLT10*, respectively. Finally, in the eight patients demonstrating a negative FG involving *KMT2A*, FISH or karyotype analyses revealed positive results for *KMT2A*-r (Patients 1, 8, 9, 10, 11, 12, 13, and 14).

## Discussion

This study simultaneously identified and described *KMT2A*-r and *KMT2A* CNV alterations in AL using FISH analysis. *KMT2A*, which is 92 kb in size, is located on chromosome 11, band 23 (11q23) and includes at least 37 exons^13^. The accurate detection of *KMT2A*-r remains a challenge in clinical practice because of the complex mechanisms involved, including reciprocal translocations, complex chromosomal rearrangements, gene internal duplications, deletions, inversions on chromosome 11q, insertion of chromatin material into *KMT2A*, and *KMT2A* insertions into other chromosomes or vice versa^14^. It has been reported that the occurrence of a 3′-*KMT2A* deletion is associated with *KMT2A*-r^15^.

Currently, the methods routinely used for detecting *KMT2A*-r in AL patients in China include CBA and RT-PCR. However, FISH analysis using a dual-color BAP is used to further confirm the existence of *KMT2A*-r when these two primary methods yield inconsistent results.

In the current study, *KMT2A* FISH was performed for samples from every patient in the AL cohort, as were CBA and RT-PCR analyses. Through a combination of the three methods, a total of 23 cases were identified as being *KMT2A*-r, with eight being positive using all three methods. Methods of FG detection, karyotype analysis, and FISH analysis yielded positive results in 15, 16, and 20 cases, respectively (Table 2). Conventional karyotype analysis, which can reveal structural and numerical abnormalities of chromosomes, may miss some instances of *KMT2A*-r, probably owing to poor metaphase division of leukemic cells and poor detection of subtle chromosomal changes caused by complex or cryptic abnormalities^6^. Use of the *LSI KMT2A* BAP allows for the recognition of complex rearrangements, notably cryptic insertions of *KMT2A* segments into other chromosomes or the disruption of *KMT2A* by the insertion of other chromosomal segments, which improves the detection rate of *KMT2A*-r^16^. Three cases in our cohort (Patients 1, 2, and 3) were confirmed to have cryptic *KMT2A* insertions after a second review of karyotype analysis, which was based on the metaphase FISH analysis of *KMT2A*. This illustrates the need for FISH analysis using the *KMT2A* probe as an important auxiliary method to the CBA of AL patients. Furthermore, beyond the typical separation of signals in FISH detection of *KMT2A*, atypical FISH signal patterns are worth studying in clinical practice, especially changes in the number and intensity of orange and green signals. When an unusual *KMT2A* signal pattern is observed, metaphase FISH should be implemented to further clarify the mechanism of complex rearrangement of *KMT2A*. Several cases of insertions involving band 11q23 into chromosomes 2, 4, 5, 6, 9, and 10 and the X-chromosome have been previously reported^16–20^.

Compared to cytogenetic analysis, RT-PCR is a more sensitive and rapid technique. Furthermore, commercially available multiplex RT-PCR kits that can reveal *KMT2A*-r with frequently involved partner genes have been used worldwide. However, these kits do not contain primer sets for detecting rare or unknown partner genes, which may result in missed diagnosis of some patients with *KMT2A*-r. The RT-PCR panel used in the present study may not have included primers to detect the FG of *KMT2A*-r caused by t(2;11), t(5;11), and t(11;17), which was demonstrated by the karyotype analysis of Patients 9, 10, 12, and 13. However, the remaining sample volumes were insufficient to further confirm the presence of this FG by RNA sequencing. Additionally, fusion transcripts cannot be found in cases with such widespread aberrations as t(4;11) (Patient 14), t(10;11) (Patient 8), or t(11;19) (Patient 11). We speculated that there are several potential causes of the negative RT-PCR result. First, in addition to the major breakpoint cluster region (BCR) of the *KMT2A* gene (*KMT2A* exons 8–14), the other BCR is novel and minor (*KMT2A* intron 21–23), which was verified by Meyer et al. in 2019^5^. The commercial multiplex RT-PCR kits are not always compatible and may only cover the major BCR, so if *KMT2A*-r occurred in minor BCR or other special mechanisms, including in the cases of 11q deletion, inversion, or three-way translocation, which can all cause *KMT2A*-r breakpoint outside of the major BCR. In these situations, the results were both beyond the range of the panel designed for the commercial kit. Second, in addition to *AFF1*, *MLLT10*, *MLLT1*, and *ELL*, different partner genes can be involved in 4q, 10p, and 19p, including *SEPT11* and *ARGBP2* (4q), *ABI1* and *NEBL* (10p), and *ASAH3*, *EEN* and *MYO1F* (19p)^4^. However, these different partner genes all demonstrate the same t(4;11), t(10;11), and t(11;19) in the karyotype, which cannot be clearly distinguished by CBA. Patients 4 and 6 showed the FG of *KMT2A*-*SEPT6* and *KMT2A*-*MLLT10* by RT-PCR without any aberration involving the rearrangement of *KMT2A* in FISH and karyotype analyses. Similar cases have been previously described by other groups, and mechanisms that involve either a fragment of *KMT2A* being inserted elsewhere into the genome or a fragment of a locus being inserted proximally to *KMT2A* have been identified^6,16^. Both circumstances create a copy-neutral *KMT2A*-fusion oncogene that is not be detectable by BAP FISH testing. In addition, *KMT2A*-PTDs that are undetectable using currently available FISH probes or conventional karyotype analysis were identified in one case of the current study (Patient 5) and confirmed by RT-PCR. Therefore, the combined use of FISH, CBA, and multiplex RT-PCR analyses can improve the detection of *KMT2A*-r, particularly in unusual, complex, or cryptic chromosomal rearrangements that are frequently observed in AL.

A total of 17 patients with *KMT2A* CNV, including 15 with AML and two with ALL, were identified in the current cohort. Moreover, 10 of 15 (66.7%) AML cases with *KMT2A* CNV detected by FISH were CK, which was higher than that for the group with normal FISH results (66.7% vs. 10.6%; Table 4). Amplification of *KMT2A* could be defined as the presence of at least two extra gene copies as seven of 10 patients with AML and CK demonstrated ≥ 4 copies of *KMT2A*. Tang et al. reported that AML/MDS with *KMT2A* amplification is associated with a CK and high frequency of *TP53* mutations^8^. It has been demonstrated by Zatkova et al. that patients with AML and CK, including 11q/*KMT2A* amplification, respond poorly to therapy and have a poor prognosis with an extremely short overall survival^9^. Therefore, the application of *KMT2A* FISH may have an additional benefit of shedding light on the identification of CK, especially when conventional karyotype analysis fails. The sample from Patient 27 of the current study failed in CBA, but FISH analysis revealed five copies of *KMT2A*. Three months after AML diagnosis, the patient had not achieved remission and died of infectious shock. Of the 10 AML cases with CK, including *KMT2A* amplification, recurrent chromosomal aberrations of 5q deletions were observed in six cases, which was similar to previous findings of 5q deletions being the most frequently associated with 11q amplifications in AML with CK^9,21^.

The mechanisms underlying the *KMT2A*-r and *KMT2A* CNV pathogenesis differ in that some *KMT2A*-r are driver abnormalities that require very few cooperate mutations to induce tumorigenesis^22^, while *KMT2A* CNV is easily accompanied by CK, meaning it must act in coordination with other genetic abnormalities to cause leukemia. Thus, detection and distinction of these two types of pathogenesis are important for development and management of treatment protocols. Several studies have demonstrated the potential use of KMT2A inhibitors as promising targeted therapies for *KMT2A*-r leukemia^23^. However, the effect on *KMT2A* CNV was not yet known at the time of this study.

Our study has several limitations. First, the commercial multiplex RT-PCR kits did not disclose primers sequences for trademark restrictions, which hindered us in distinguishing whether there were some defects in their primer design leading to the false negative in our cohort. Second, because of the insufficient sample and unaffordable cost, the cases with discrepant results from the three detection techniques cannot be further clarified using the complex mechanism of *KMT2A*-r by taking long distance inverse PCR (LDI-PCR) or next generation sequencing (NGS) technologies, such as RNA sequencing, which were both previously considered as robust ways to identify *KMT2A*-r.

Despite these limitations, we have shown that using multiplex nested RT-PCR, karyotype analysis, and FISH techniques in combination can improve the detection rate of *KMT2A*-r. We think the results show that *KMT2A* FISH detection should become a routine component of diagnostic and prognostic workups to identify cryptic *KMT2A*-r in AL patients. Furthermore, analysis may reveal a set of different *KMT2A* CNVs, which is highly associated with poor prognosis in AL, especially for patients in which there is a failure to obtain adequate levels of evaluable metaphase cells in cytogenetic analysis.

## Data Availability

The datasets used and analyzed during the current study are available from the corresponding author on reasonable request.
